# Hyper high haemoglobin content in red blood cells and erythropoietic transitions postnatally in infants of 22 to 26 weeks’ gestation: a prospective cohort study

**DOI:** 10.1136/archdischild-2022-325248

**Published:** 2023-05-11

**Authors:** Sara Marie Larsson, Tommy Ulinder, Alexander Rakow, Mireille Vanpee, Dirk Wackernagel, Karin Sävman, Ingrid Hansen-Pupp, Ann Hellström, David Ley, Ola Andersson

**Affiliations:** 1 Department of Clinical Sciences Lund, Paediatrics, Lund University, Lund, Sweden; 2 Department of Clinical Chemistry, Hospital of Halland, Varberg/Halmstad, Sweden; 3 Department of Neonatology, Skåne University Hospital, Lund/Malmö, Sweden; 4 Department of Women’s and Children’s Health, Karolinska Institutet and Karolinska University Hospital, Stockholm, Sweden; 5 Department for Clinical Science, Intervention and Technology (CLINTEC), Karolinska Institutet, Stockholm, Sweden; 6 Department of Neonatology, Johannes von Gutenberg University, Mainz, Germany; 7 Department of Paediatrics, Sahlgrenska Academy, Gothenburg, Sweden; 8 The Sahlgrenska Centre for Paediatric Ophtalmology Research, Department of Clinical Neuroscience, Sahlgrenska Academy, Gothenburg, Sweden

**Keywords:** haematology, intensive care units, neonatal, neonatology, growth

## Abstract

**Objective:**

Blood cell populations, including red blood cells (RBC) unique to the extremely preterm (EPT) infant, are potentially lost due to frequent clinical blood sampling during neonatal intensive care. Currently, neonatal RBC population heterogeneity is not described by measurement of total haemoglobin or haematocrit. We therefore aimed to describe a subpopulation of large RBCs with hyper high haemoglobin content, >49 pg (Hyper-He) following EPT birth.

**Design:**

Prospective observational cohort study.

**Setting:**

Two Swedish study centres.

**Participants:**

Infants (n=62) born between gestational weeks 22^+0^ to 26^+6^.

**Methods:**

Prospective data (n=280) were collected from March 2020 to September 2022 as part of an ongoing randomised controlled trial. Blood was sampled from the umbilical cord, at postnatal day 1–14, 1 month, 40 weeks’ postmenstrual age and at 3 months’ corrected age.

**Results:**

At birth, there was a considerable inter-individual variation; Hyper-He ranging from 1.5% to 24.9% (median 7.0%). An inverse association with birth weight and gestational age was observed; Spearman’s rho (CI) −0.38 (−0.63 to −0.07) and −0.39 (−0.65 to −0.05), respectively. Overall, Hyper-He rapidly decreased, only 0.6%–5.0% (median 2.2%) remaining 2 weeks postnatally. Adult levels (<1%) were reached at corresponding term age.

**Conclusion:**

Our results point to gestational age and birth weight-dependent properties of the RBC population. Future work needs to verify results by different measurement techniques and elucidate the potential role of differing properties between endogenous and transfused RBCs in relation to neonatal morbidities during this important time frame of child development.

**Trial registration number:**

NCT04239690.

**WHAT IS ALREADY KNOWN ON THIS TOPIC**

- Erythropoietic contributions from different physiological and cellular origins may contribute to a heterogeneity in the peripheral red blood cell (RBC) population during 22 to 26 weeks’ gestation.

**WHAT THIS STUDY ADDS**

- The proportion of neonatal RBCs with hyper high haemoglobin content (Hyper-He) decreases rapidly during first weeks in neonatal intensive care.
- Proportions of Hyper-He measured early after birth could be indicative of erythropoietic transitions and/or stress erythropoiesis and are associated with gestational age as well as birth weight.

**HOW THIS STUDY MIGHT AFFECT RESEARCH, PRACTICE OR POLICY**

- The role that the decrease of characteristic endogenous neonatal RBCs plays for oxygenation, perfusion and the development of neonatal morbidities in EPT infants is not well understood. Future studies addressing these knowledge gaps are warranted.

## Introduction

Globally, preterm birth affects 15 million newborns each year.^1^ Although survival rates increase with modern neonatal care, major challenges remain, particularly for the most preterm born infants.^2–4^ During initial care, these infants have one of the highest transfusion requirements within hospital settings, as the majority develop anaemia that could compromise oxygenation of vital organs. The aetiology of this neonatal anaemia is multi-factorial, but explanatory models often address decreasing erythropoietin levels following transition from a relatively hypoxic intrauterine environment to more oxygen-rich conditions after birth.^5 6^ Also well-known is a considerable blood volume loss related to frequent clinical blood sampling^7^ causing a potential depletion of unique endogenous red blood cells (RBCs).^8 9^

In contrast to the RBCs from the adult definitive erythroid lineage, originating from bone marrow and haematopoietic stem cells (HSC), the liver is the main erythropoietic organ during the gestational period ascribed to extremely preterm (EPT) birth.^10^ Until recently, it was assumed that HSC drive fetal liver erythropoiesis already from 10th to 12th gestational weeks onwards.^11 12^ As new lineage-tracing experiments in murine models however suggest HSC-independent pathways to be present as well as the continuing contribution of erythromyeloid progenitors (EMP) to liver erythropoiesis during fetal life, these are results that may have implications also on the general understanding of human fetal and infant erythropoiesis.^13 14^

A gradual process where the HSC definitive erythroid lineage transitions to the more protected environment in the bone marrow is initiated at 16th to 18th week of gestation.^15^ During later gestational weeks, corresponding to the period of EPT birth, the actual extent of different possible erythropoietic contributions remains to be clarified.^16–18^

With potentially different physiological, microenvironmental and cellular origins, the resulting peripheral RBC population may not be homogeneous, potentially having different properties between subpopulations. The mean cell haemoglobin concentration index (MCHC) after EPT birth is similar to adult values,^19^ but the RBC count expected at these gestational weeks is essentially lower.^20^ This implies that either each RBC transports more haemoglobin or that haemoglobin is unevenly distributed in heterogeneous subpopulations of red cells, such as large red cells with hyper high haemoglobin content (Hyper-He). The proportion of cells with a particularly high haemoglobin content in adults can be estimated based on the measurement of high-angle forward scatter on Sysmex XN haematology instruments (Hyper-He, >49 pg/cell). While Hyper-He is low in healthy adult blood, <1%,^21^ little is known about proportions in newborns.

Our objective of this study was to describe proportions of Hyper-He following EPT birth as a potential biomarker of an erythropoietic transitional process. We also aimed to investigate the influence of gestational age, birth weight and fetal growth on early proportions of Hyper-He.

## Methods

### Study population

At birth, the infants had a gestational age of 22^+0^ to 26^+6^ weeks. Clinical data were obtained from clinical records. Fetal growth restriction (FGR) was diagnosed in cases with abnormal fetal Doppler velocimetry combined with estimated fetal weight deviation^22^ and a birth weight SD score below –2 SD calculated according to Niklasson and Albertsson-Wikland.^23^ Exclusion criteria was major malformations. Data were collected during March 2020 to September 2022 as part of an ongoing two-armed multicentre randomised controlled intervention trial. The aim of the main trial is to investigate the relationship between preservation of neonatal blood factors and neonatal morbidity using blood sampling micro-methods. Our ancillary observational cohort study was carried out at the neonatal intensive care units at the Southern Region University Hospital, Lund, and Karolinska University Hospital, Stockholm, in Sweden.

### Specimen collection and handling

Blood samples were collected at birth (umbilical cord blood) and thereafter through an umbilical catheter, peripheral arterial catheter or peripheral venous puncture. During postnatal day 1–14, only blood from clinical sampling was used to analyse the RBC data presented in this study thereby not causing any additional blood loss, essential for the main study design. Blood (250 µL) was collected in microtainer EDTA tubes (BD Vacutainer, Plymouth, UK). In addition to the clinical samples, study samples were obtained at postnatal age 1 month (median age 27 days, IQR 26–29 days, min 21, max 41 days), at postmenstrual age week 40 and at 3 months corrected age. All blood samples were promptly sent to the hospital laboratory for analysis to avoid RBC swelling. Samples were analysed for Hyper-He (proportion of cells with haemoglobin >49 pg/cell), haemoglobin concentration (Hb), mean cell haemoglobin content (MCH), mean cell volume (MCV) and reticulocyte counts (RET).

### Laboratory analysis

Blood was analysed on Sysmex XN (Sysmex, Kobe, Japan) at the clinical laboratories at Lund University Hospital and at Karolinska University Hospital. The instrument uses laser to measure forward and side scatter light on a cell-by-cell basis. Measurements of RBC count are based on impedance. Total haemoglobin concentration is measured by an absorption photometric method. The index MCH is derived by the ratio haemoglobin/RBC count and MCV by the ratio haematocrit/RBC count. The instruments were under regular quality control for commonly used haematological parameters and laboratories were accredited according to ISO15189.

### Calculations and statistical analysis

Data are presented as mean (±SD), median (IQR), range (min – max) or number (%) where appropriate. Correlations were estimated using Spearman’s rho where p values <0.05 were considered statistically significant. SPSS V.27.0 (IBM, SPSS) was used for data analysis.

## Results

### Study cohort

**Figure 1:**
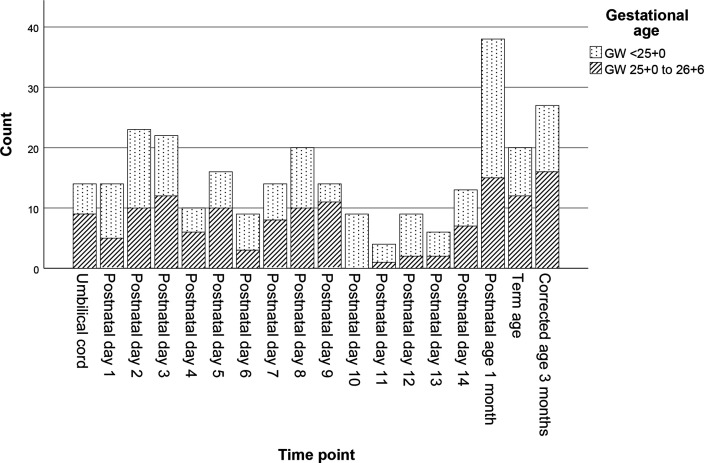
Number of measurements for each sampling time point and proportion of gestational ages below and above 25 weeks, respectively.

Blood measurements (n=280) were available from 62 of 73 infants enrolled in the randomised controlled trial at the two neonatal intensive care units during the present time period. The mean (SD) birth weight of the 62 infants was 727 g (171 g) and median gestational age at birth 24^+4^. Gestational ages and clinical characteristics are presented in table 1. Distribution in number of measurements, with regard to sampling time point and gestational ages, is shown in figure 1.

**Table 1:** Participant characteristics

Gestational age at birth (weeks+days)	Infants (% of total n=62)
22+0 to 22+6	3 (5)
23+0 to 23+6	12 (19)
24+0 to 24+6	13 (21)
25+0 to 25+6	16 (26)
26+0 to 26+6	18 (29)
Antenatal steroid treatment	60 (97)
Preeclampsia	4 (6)
Chorioamnionitis	17 (27)
Fetal growth restriction	10 (16)
Male	33 (53)
Multiple gestation	22 (35)

### RBCs with Hyper-He during the first 14 postnatal days

**Figure 2:**
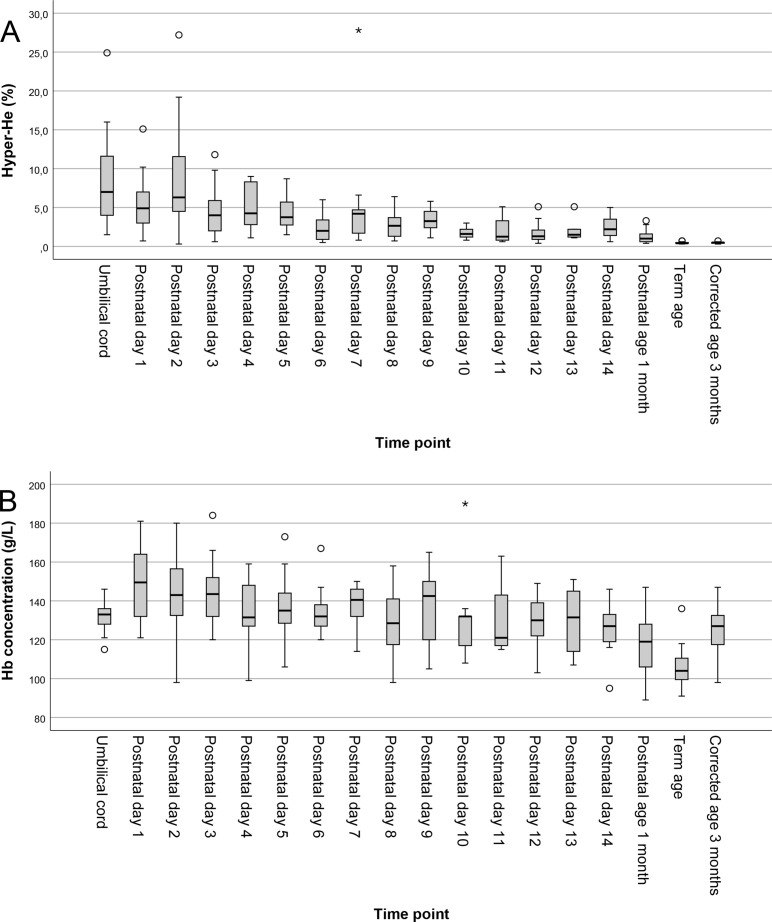
Box-plots showing longitudinal trends of the proportion of Hyper-He (A) and haemoglobin concentration (B) in the blood of extremely preterm born infants (n=62) cord blood, postnatal day 1–14, at 1 month postnatal age, postmenstrual age week 40 and at corrected age 3 months.

Umbilical cord samples (n=15) were analysed. One sample with an outlier Hb result (57 g/L) was excluded due to suspected measurement interference. In the remaining samples (n=14), there was a large inter-individual variability in Hyper-He ranging from 1.5% to 24.9% (median 7.0%). Association between Hyper-He and RET in the umbilical cord blood was not statistically significant but pointed towards a possible weak inverse association, Spearman’s rho −0.47 (CI −0.81 to 0.10), p<0.09. Overall, the median proportion of Hyper-He rapidly decreased with increasing postnatal age, at postnatal day 14 reaching 2.2% (0.6%–5.0%) and by term age adult levels <1%. The corresponding total haemoglobin concentration in cord samples was median 133 g/L (115–146 g/L), slightly decreasing to 127 g/L (95–146 g/L) at postnatal day 14. The lowest median Hb concentration was observed at term age, 104 g/L (91–136 g/L). Figure 2 presents box-plots for Hyper-He and haemoglobin at each time point.

### Association between Hyper-He and the indices MCH and MCV

**Figure 3:**
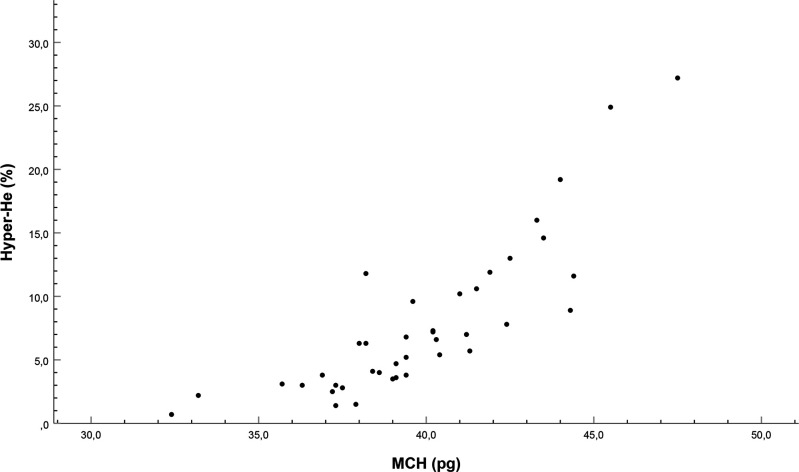
A high proportion of Hyper-He early after birth (<48 hours) is associated with a high mean cell content of haemoglobin (MCH) as derived from measured concentration of total haemoglobin (photometry) and red blood cell count (impedance).

Strong associations were found between the proportion of Hyper-He (optically measured) and the mean haemoglobin content of the red cells, MCH (derived from photometrical measurement of Hb divided by RBC count measured by impedance) as well as MCV (derived from haematocrit and RBC count). As for MCH, Spearman’s rho was 0.87 (CI 0.77 to 0.93), p<0.001 (figure 3). The association of Hyper-He with MCV was similar in magnitude, Spearman’s rho 0.86 (CI 0.75 to 0.93), p<0.001.

As by ratio, a large MCH indicates higher total haemoglobin distributed over a lower number of RBC counts, this could mean that infants having a high proportion of Hyper-He at birth also overall have haemoglobin accumulated in fewer, larger cells. The 2.5th percentile and 97.5th percentile for MCH were 32 and 47 pg, respectively. Corresponding percentiles for MCV were 99 and 139 fL.

### Postmenstrual age, birth weight and FGR in relation to the proportion of Hyper-He at birth

**Figure 4:**
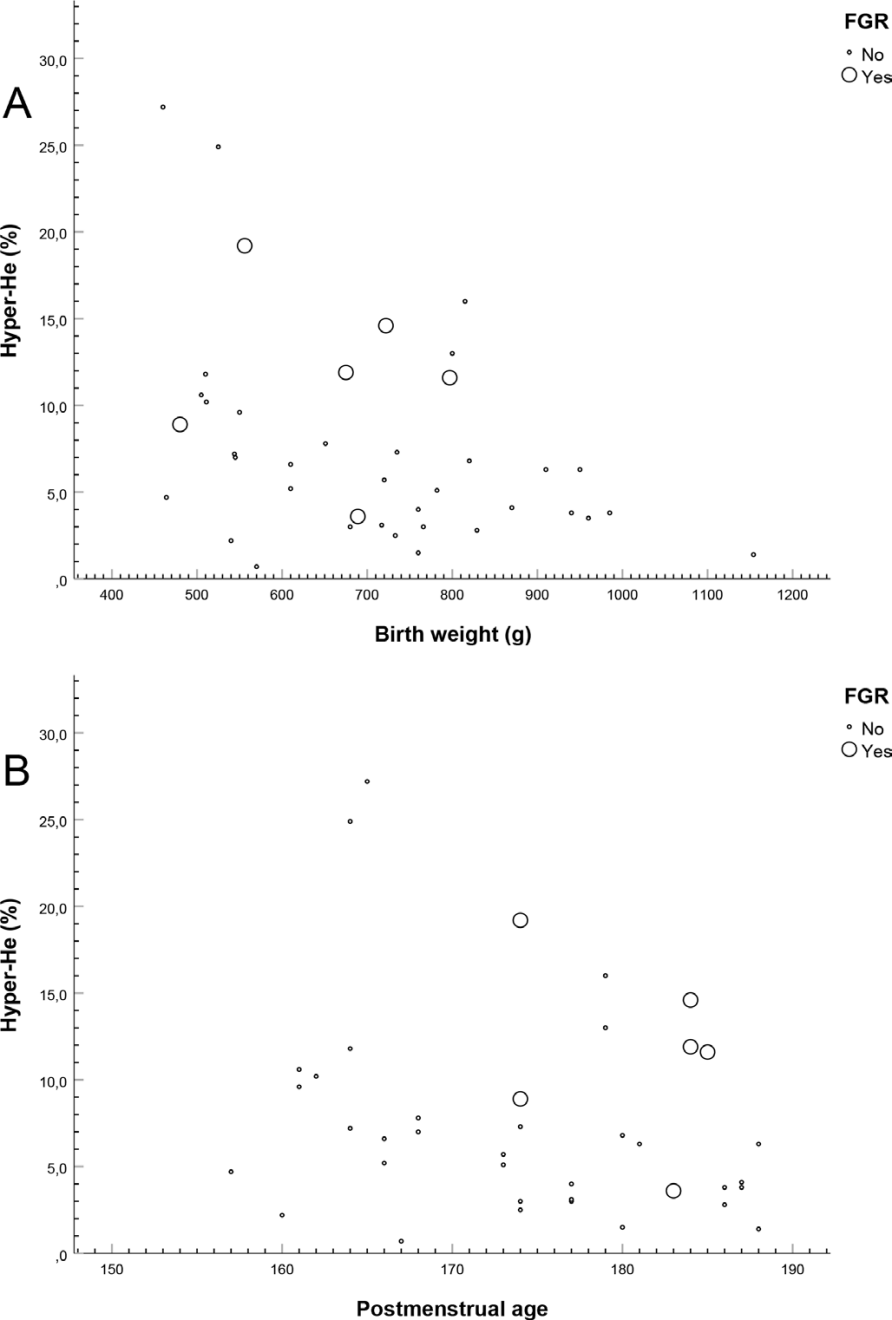
The associations between birth weight (A) and postmenstrual age (B), respectively, and the proportion of Hyper-He measured within 48 hours after birth. FGR, fetal growth restriction (defined by abnormal fetal Doppler velocimetry combined with estimated fetal weight deviation and birth weight SD score).

For 40 infants, Hyper-He was measured within 48 hours. There was a statistically significant inverse association between birth weight and Hyper-He, Spearman’s rho −0.38 (CI −0.63 to −0.07), p=0.02. Also, if the infants with FGR (abnormal fetal Doppler velocimetry combined with estimated fetal weight deviation and a birth weight SD score, in total n=6) were excluded (n=34), we found an association of similar magnitude with regard to gestational age, Spearman’s rho −0.39 (CI −0.65 to −0.05), p=0.02, figure 4. The infants with FGR had median Hyper-He; 11.8% (IQR 8.9%–14.6%) compared with 5.5% (IQR 3.1%–8.3%) for infants without FGR.

## Discussion

We report the measurement of a subpopulation of RBCs with a particularly high content of haemoglobin (>49 pg) in EPT infants. Early after birth, the proportion of these cells is inversely associated with birth weight as well as gestational age and varies substantially between individuals. Also, a rapid decrease postnatally is observed.

Development of anaemia after EPT birth has so far mainly been attributed to the decreasing erythropoietin levels following birth. This study adds further aspects to this model.

First, as individual variation during the first postnatal days was considerable (2%–25%), we hypothesise that infants with a high proportion of Hyper-He at birth are more at risk for severe nadirs in Hb, as this cell population mainly seems to be lost reflected by the considerable decrease during the first 14 postnatal days. Avoiding loss of large endogenous RBCs by minimising clinical blood sampling volumes might therefore be of particular importance for these infants.

Second, although the origin of these cells is currently unknown, their presence as well as prolonged raised values might suggest a general transitional delay in the establishment of definitive HSC erythropoietic lineage erythropoiesis. The Hyper-He RBCs could be a result from stress erythropoiesis,^24 25^ but there are also pre-clinical reports describing large enucleated RBCs of primitive origin, even after term birth.^26^ Erythroid progenitors of EMP lineage have been found to differ in responsiveness to erythropoietin stimulation compared with HSC lineage.^13^

Our current knowledge of the neonatal red cell population in clinical care has so far mostly been limited to describing the RBC population as homogeneous, using parameters such as total Hb, Hct and mainly derived indices such as MCHC (ratio of Hb and haematocrit) and MCH (ratio of total Hb and RBC). The 2.5th to 97.5th interval of MCH estimated in our study was 32 to 47 pg during the first postnatal days. The 97.5th percentile was thereby higher than previously reported for infants of the same gestational age (33 to 41 pg).^11^ In term infants, MCH is substantially lower; 32 to 38 pg^27^ and adult MCH is only 27 to 33 pg/cell.^28^ Our results indicate that haemoglobin is accumulated in fewer larger cells with higher Hb content in infants with a high proportion of Hyper-He.

With regard to fetal growth, we observed that infants born after FGR seemed to have a high proportion of Hyper-He early after birth, suggesting a positive relationship between chronic fetal hypoxia and proportion of Hyper-He cells. Reibel *et al* 29 described higher transfusion rates during the first week of life in EPT born neonates following FGR with initially similar haemoglobin concentrations. This could indicate the potential dependency on fewer high-loaded Hb cells for oxygen transport as well as presence of stress erythropoiesis. High Epo concentrations have previously been reported in the cord blood of preterm newborns subject to chronic fetal stress, but Epo levels have not been related to Hb or haematocrit.^30^ Neither have Epo concentrations been correlated to a response in RET.^31^ The results from our study therefore suggest that the erythropoietic response to Epo stimulation could include an increase in a subpopulation of few but large red cells with increased Hb content. This hypothesis however needs to be investigated in future studies.

Strengths of our current study include utilisation of the blood from clinical sampling for measurement of the RBC population, thereby requiring no extra phlebotomies day 1–14. Samples were promptly transported to the laboratory and thus RBC swelling was avoided. This is also a prospectively studied, longitudinally followed cohort.

We also identify several weaknesses, and our results do need verification by other methodologies. Mainly, these instruments and algorithms are developed for adults and for children at older ages than our cohort. The extent to which they are verified for the blood from the most preterm infants is currently unknown, thereby introducing analytical uncertainty. The risk for interferences or misclassification of cells cannot be ruled out. Yet, we observed excellent repeatability as well as a strong association with MCH, an index being derived from different measurement techniques in the instrument. An additional drawback to this study is a bias towards more critically ill infants during postnatal day 1–14 due to the blood sampling scheme. Neither does the current study differentiate between a possible dilution effect resulting from pRBC transfusions to an actual loss of these cells due to intrinsic or extrinsic factors. This also needs to be addressed in future studies.

Further, with regard to antenatal steroids, these may have influenced the results stimulating erythropoiesis.^32^ Of the infants, 97% were born after antenatal steroid treatment which limited the possibilities to investigate this variable further. The size of our study population was also too small to study the impact of chorioamnionitis on Hyper-He.

Endogenous neonatal RBCs could hypothetically play an important role during this critical time frame for future development. Haematopoietic observations that differ from adults, that historically have been described as ‘immature’ are now beginning to be more widely understood as intricate pathways unique to fetuses and neonates allowing to populate the bone marrow and concurrently meet the needs for increasing blood volumes following rapid growth.^33^ It can be hypothesised that preserving large high haemoglobin content cells in circulation during the transition to an established definitive infant erythropoiesis could impact haemodynamics; gas diffusion properties may act in concert with fetal haemoglobin. These aspects related to oxygenation need further investigation.

In conclusion, our hypothesis generating study implies a possible heterogeneity in the endogenous RBC population after EPT birth where high proportions of peripheral large hyper haemoglobin-loaded RBCs might be indicative of an erythropoietic transitional process. Future work needs to verify results by different measurement techniques. Further investigations regarding endogenous RBCs in relation to sampling-related blood loss, development of anaemia, transfusions as well as neonatal morbidities are warranted.

## Data Availability

Data are available upon reasonable request. The data are not publicly available due to privacy or ethical restrictions.
